# An attraction–repulsion transition of force on two asymmetric wedges induced by active particles

**DOI:** 10.1038/s41598-020-68677-w

**Published:** 2020-07-16

**Authors:** Ke Li, Fuchen Guo, Xiaolin Zhou, Xianghong Wang, Linli He, Linxi Zhang

**Affiliations:** 1 Department of Physics, Zhejiang University, Hangzhou, 310027 Zhejiang China; 2 Department of Physics, Wenzhou University, Wenzhou, 325035 Zhejiang China

**Keywords:** Biophysics, Energy science and technology, Physics

## Abstract

Effective interaction between two asymmetric wedges immersed in a two-dimensional active bath is investigated by computer simulations. The attraction–repulsion transition of effective force between two asymmetric wedges is subjected to the relative position of two wedges, the wedge-to-wedge distance, the active particle density, as well as the apex angle of two wedges. By exchanging the position of the two asymmetric wedges in an active bath, firstly a simple attraction–repulsion transition of effective force occurs, completely different from passive Brownian particles. Secondly the transition of effective force is symmetric for the long-range distance between two asymmetric wedges, while it is asymmetric for the short-range case. Our investigations may provide new possibilities to govern the motion and assembly of microscopic objects by taking advantage of the self-driven behaviour of active particles.

## Introduction

Differently from passive Brownian particles, active particles (also known as self-propelled particles) may acquire energy from external source and drive themselves far away from equilibrium. Examples of active matter can be found in various systems ranging from bacteria^1,2^, alga^3,4^, spermatozoa^5,6^, macroscopic animals^7,8^ to artificial micro-swimmers^9–12^. Meanwhile active particles exhibit spatiotemporal active states, such as swarming^13–15^, and turbulence(swirling)^16–18^. Therefore, researches on the nonequilibrium dynamics of active particles have great significance in not only clarifying its physical mechanisms^19–23^ but also understanding the natural biological systems, such as bacterial colonies, fish schools, bird flocks^24^ and so on. Additionally, active particles with a proper design can be widely applied in a variety of areas, such as drug delivery in medicine^25,26^, gene therapy^27^, nanoscale assembly^28,29^, pollution management^30^, etc.

Passive objects or rigid obstacles immersed into an active bath can guide and accumulate active particles^31–34^, as well as be taken as trapping devices for active swimmers^35–37^. Conversely, active swimmers placed by fixed boundaries would undergo a rectification^38,39^ and sorting^40–42^ effect. Perhaps the most remarkable phenomenon is that active particles tend to accumulate at the fixed boundaries due to the reduction of its mobility in the proximity of the fixed boundaries. This creates positive feedback corresponding to the motility-induced phase separation (MIPS) between dense and dilute fluid^43–50^: active particles accumulate where they move more slowly, while steric hindrance or repulsive interactions slow down active particles at high density. Solon et al*.* successfully introduced an effective free-energy to describe MIPS as a spinodal decomposition in terms of density-field^48^. MIPS can lead to cohesive active matter in the absence of cohesive forces and the phase-coexistence of active particles displays several relevant differences from the liquid–gas phase coexistence of passive particles^49,50^. Sandal et al*.* found that active particles can self-organize into two coexisting phase at different kinetic temperatures, and they are separated from each other by a sharp and persistent temperature gradient^50^. Ray et al*.* observed that a robust attractive force appears between two parallel rods immersed into a bath of run-and-tumble active particles, which results from a depletion of active particles in the confinement between two rods by the combined effect both particles swimming along the rods and a geometric shadowing effect. Their results also give a fact that objects can experience a great variety of fluctuation-induced forces in active particles system^33^. Kaiser et al*.* showed that a static chevron-shape boundary would represent an excellent trapping device for active particles, and the trap efficiency behaves a phase transition corresponding to three states: partial trapping, complete trapping, no trapping, which is determined by the apex angle of the trap^36^. Kaiser et al*.* also experimentally focused on the motion of a pair of symmetrical micro-wedges submersed in a turbulent rod-like bacteria bath. They observed that two micro-wedges of same orientation move such that their mutual distance decreases, while they drift apart for an anti-parallel orientation^37^. In our recent work^43^, we explored the collective behaviours of active rod-like particles in two symmetrical micro-wedges of anti-parallel orientation using computer simulations. A transition from repulsion to attraction occurs by varying both apex angles of two wedges, which is also sensitive to the particle density. The optimal apex angle θ* and particle density ρ* is characterized by the saturated trapping of active particles inside wedge^43^.

Due to the intrinsic non-equilibrium property of active particles, it is interesting to study the non-equilibrium behaviours of active swimmers in a system immersed by an array of asymmetric barriers^1,51–58^. The motion of active particles can be rectified in the presence of funnel-shaped obstacles and a ratchet effect is observed even in the absence of an external drive^56,57^. Volpe et al*.*^53^ found that active swimmers would be sorted with the use of a periodic array of convex obstacles (ellipses). According to these investigations, the asymmetry of obstacles also plays a crucial role in the occurrence of these phenomena, which originates from the broken time symmetry^53^ in particle-obstacle interactions. In fact, as we know, in active particle system, the ratchet phenomenon demands two ingredients^38^ which are (a) fluctuating input zero-mean force: it should break the thermodynamical equilibrium, and (b) asymmetry (temporal and/or spatial): it can violate the left–right symmetry of the response. In this paper, two asymmetric wedges are immersed in a bath of active rod-like particles, which are taken as the trap device, and our aim is to investigate some special non-equilibrium properties of asymmetric obstacles in an active particle bath. We monitor the directed collective behaviour of active particles near the wedges with variable wedge-to-wedge distance, active particle density, and apex angles of wedge. The final focus is on the attraction–repulsion transition of effective force between two asymmetric wedges. By exchanging the position of the two asymmetric wedges, the attraction–repulsion transition of effective force occurs and this transition is symmetrical at long-range distance, while it is asymmetrical at short-range distance.

## Model and methods

**Figure 1:**
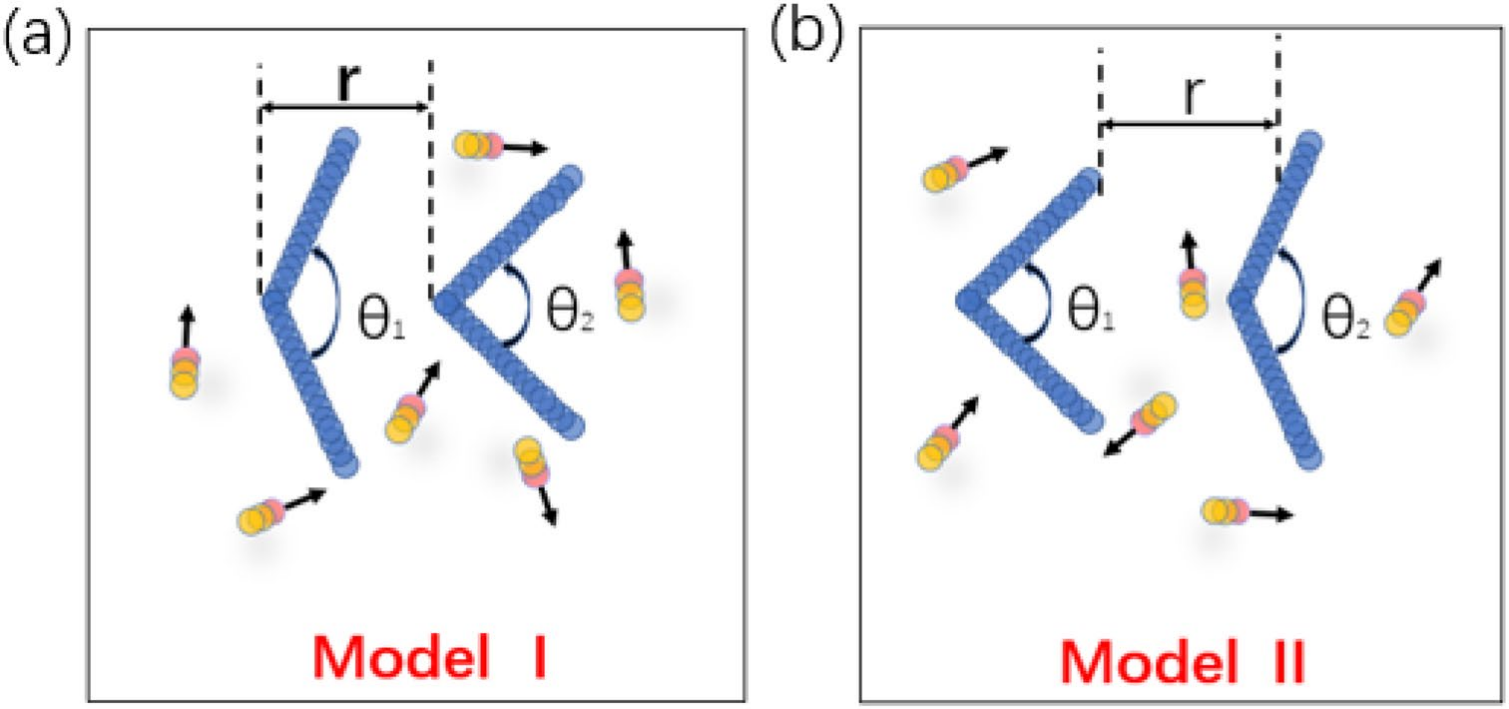
Simple model of two asymmetric wedges in a 2D active bath. r represents the x-direction nearest distance between the two wedges and the apex angles of wedges are denoted by θ_1_ and θ_2_. The active rod-like particles are shown in yellow, where the head of the particles is highlighted by red color. Two types of wedges are considered: (**a**) Model I (θ_1_ = 165° and θ_2_ = 90°) and (**b**) Model II (θ_1_ = 90° and θ_2_ = 165°), respectively.

In our simulation, two asymmetric hard wedges in a suspension of a two-dimensional active bath are considered, shown in Fig. 1^37,43^. The active bath is modelled by N rod-like self-propelled particles. Each rod-like particle with length l = σ and width d = 0.5σ consists of three spherical beads, which are equidistantly positioned with a displacement S = 0.25σ, along the main rod axis^37,43^. Here σ is the reduced unit of length. The particle density ρ is defined by the ratio of the area occupied by N self-propelled particles to the total area of the system, which can be freely controlled by varying N. The related aspect ratio of the active particle is denoted as p = l/d (i.e., the length and width of the active particle are l and d, respectively), and here p = 2.0, which is the same as that considered in our previous work^43^. Initially, active rod-like particles are placed randomly in the system. Simulations are performed on the boxes of size L_x_ = 200σ and L_y_ = 100σ with Periodic Boundary Condition in x- and y-directions, and two hard wedges are embedded in the active bath. We focus on the case of two wedges with same orientation of different apex angles, θ_1_ and θ_2_ (varying from 0° to 180°), particle density, ρ, and distance, r. Here r is defined as the nearest distance between two wedges in x direction, which is not the distance between two cusps of wedges shown in Fig. 1b. Each wedge with length L = 20.5σ and width d = 0.5σ is discretized into 81 spherical beads equidistantly distributed, with a displacement S = 0.25σ.

Any overlap of particle–particle or particle-wedge is prohibited by choosing a shifted and cut-off Lennard–Jones potential,

1 $$ U\left( R \right) = \left\{ {\begin{array}{*{20}c} {4\varepsilon \left[ {\left( {\frac{\sigma }{R}} \right)^{12} - \left( {\frac{\sigma }{R}} \right)^{6} + \frac{1}{4}} \right] R \le 2^{\frac{1}{6}} \sigma } \\ { 0 R > 2^{\frac{1}{6}} \sigma ,} \\ \end{array} } \right. $$

where R is the distance between any two beads (particle–particle or particle-wedge), and ε = 1.0k_B_T. Here k_B_ is the Boltzmann constant and T is the temperature. Active particles swim in a low Reynolds number regime, and the dynamics of the *i*-th active particle is described by the translational velocity $v_{i}$ and the angular velocity $w_{i}$, obeying the following equtions^43,59–61^

2 $$ v_{i} = M_{i} F_{i} , $$

3 $$ w_{i} = K_{i} T_{i} , $$

where

4 $$ M_{i} = m_{\parallel } \hat{e}_{i} \hat{e}_{i} + m_{ \bot } \left( {E_{3} - \hat{e}_{i} \hat{e}_{i} } \right), $$

5 $$ K_{i} = k_{\parallel } \hat{e}_{i} \hat{e}_{i} + k_{ \bot } \left( {E_{3} - \hat{e}_{i} \hat{e}_{i} } \right) $$

are the translational and rotational mobility matrices, respectively, and *E*_3_ represents the identity matrix. In planar geometry, $k_{\parallel }$ has no contribution in the equations of motion, and mobility value for $k_{ \bot }$ is set at 4.8. Translational diffusion coefficient $m_{ \bot }$ is subjected to the aspect ratio of particle p, and here $m_{ \bot }$ = 0.8452 for p = 2^43,48,62–64^. The self-propulsion contribution to the total force $F_{i}$ is $f_{0} \hat{e}\left( {1 - \theta_{i} } \right)$, where $f_{0}$ corresponds to the magnitude of the self-driving force and θ_i_ represents the dichotomous state variable, which turns from the value 0 to 1 with the rate λ stochastically. Here, θ_i_ = 0 and θ_i_ = 1 are indicated as two states, corresponding to the running state and the tumbling state, respectively^59,60^. During the tumbling state, the total torque $T_{i}$ receives a random contribution $T_{i} \theta_{i}$ that changes the free swimming direction of the active particles^59,60^, and the tumbling rate is fixed at λ = 0.1. In our simulation, all quantities are reported in reduced units (σ = 1, $m_{\parallel }$ = 1, k_B_T = 1, and ${ }f_{0}$ = 1) and are chosen to be the units of length, mass, energy, and force, respectively. The time step is Δt = 10^–4^, and each simulation runs from 5 × 10^7^ up to a maximum of 10^8^ steps. To identify the steady-state, we average the relevant physical quantities over exponentially increasing time windows, and we use this time binning procedure to assess convergence to the steady state^65^. All statistical properties are averaged over 50 runs. Generally, the magnitude of the self-driving force is $f_{0}$ = 1.0 except special declaration.

The effective average forces F(*r*) between the two wedges immersed in the active bath are calculated by the total forces of the wedges exerted by the active particles. Here we only consider the horizontal direction average effective force because of the symmetry of the upper and lower parts of each wedge. The effective forces F(r) > 0, F(r) < 0, and F(r) = 0 represent three interaction states between two asymmetric wedges, corresponding to repulsion, attraction and no interaction, respectively.

## Results and discussion

### Attraction–repulsion transition for asymmetric wedges

**Figure 2:**
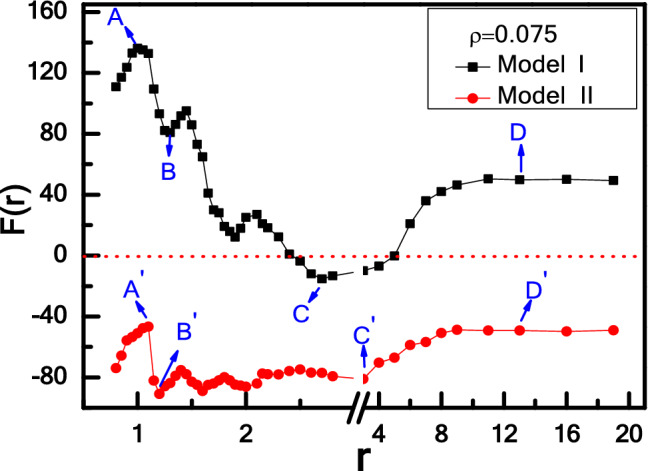
Effective force F(r) between two wedges as a function of r for Model I- and II-wedges.

We first investigate the effective force F(r) between two asymmetric wedges as a function of r at a fixed particle density of ρ = 0.075, considering two types of wedges, i.e., Model I (θ_1_ = 165° and θ_2_ = 90°) and Model II (θ_1_ = 90° and θ_2_ = 165°), as shown in Fig. 2. The interactions of active particles with the wedges are accounted through the potential U_total_ = U_1_(${\text{r}}_{{\text{i}}} - {\text{r}}_{{{\text{Li}}}}$) + U_2_(${\text{r}}_{{\text{i}}} - {\text{r}}_{{{\text{Ri}}}}$), where U_1_ and U_2_ are the contributions from the left and right wedges, which are given by Eq. (1), respectively. r_Li_ and r_Ri_ are the points located long the longitudinal axis of the left and right wedges that are closest to the i-th active particles, respectively^62^. Therefore, the effective force F(r) is the relative force between two wedges, which is defined as^63^

6 $$ {\text{F}}\left( {\text{r}} \right) = {\text{F}}_{{{12}}} - {\text{F}}_{{{34}}} . $$

F_12_ is denoted as the force exerted by active particles on the left wedge, which consists of two parts, i.e.,

7 $$ {\text{F}}_{{{12}}} = {\text{F}}_{{2}} - {\text{F}}_{{1}} $$

with

8 $$ {\text{F}}_{{1}} = \left| {\mathop \sum \limits_{{{\text{i}} \in {\text{O}}\_{\text{l}}}}^{{}} \nabla_{{{\text{x}}_{{\text{i}}} }} {\text{U}}_{1} \left( {{\text{r}}_{{\text{i}}} - {\text{r}}_{{{\text{Li}}}} } \right)} \right|, $$

9 $$ {\text{F}}_{{2}} = \left| {\mathop \sum \limits_{{{\text{i}} \in {\text{l}}\_{\text{O}}}}^{{}} \nabla_{{{\text{x}}_{{\text{i}}} }} {\text{U}}_{1} \left( {{\text{r}}_{{\text{i}}} - {\text{r}}_{{{\text{Li}}}} } \right)} \right|. $$

A similar definition for F_34_ is given by

10 $$ {\text{F}}_{{{34}}} = {\text{F}}_{{4}} - {\text{F}}_{{3}} $$

with

11 $$ {\text{F}}_{{3}} = \left| {\mathop \sum \limits_{{{\text{i}} \in {\text{O}}\_{\text{r}}}}^{{}} \nabla_{{{\text{x}}_{{\text{i}}} }} {\text{U}}_{2} \left( {{\text{r}}_{{\text{i}}} - {\text{r}}_{{{\text{Ri}}}} } \right)} \right|, $$

12 $$ {\text{F}}_{{4}} = \left| {\mathop \sum \limits_{{{\text{i}} \in {\text{r}}\_{\text{O}}}}^{{}} \nabla_{{{\text{x}}_{{\text{i}}} }} {\text{U}}_{2} \left( {{\text{r}}_{{\text{i}}} - {\text{r}}_{{{\text{Ri}}}} } \right)} \right|. $$

**Figure 3:**
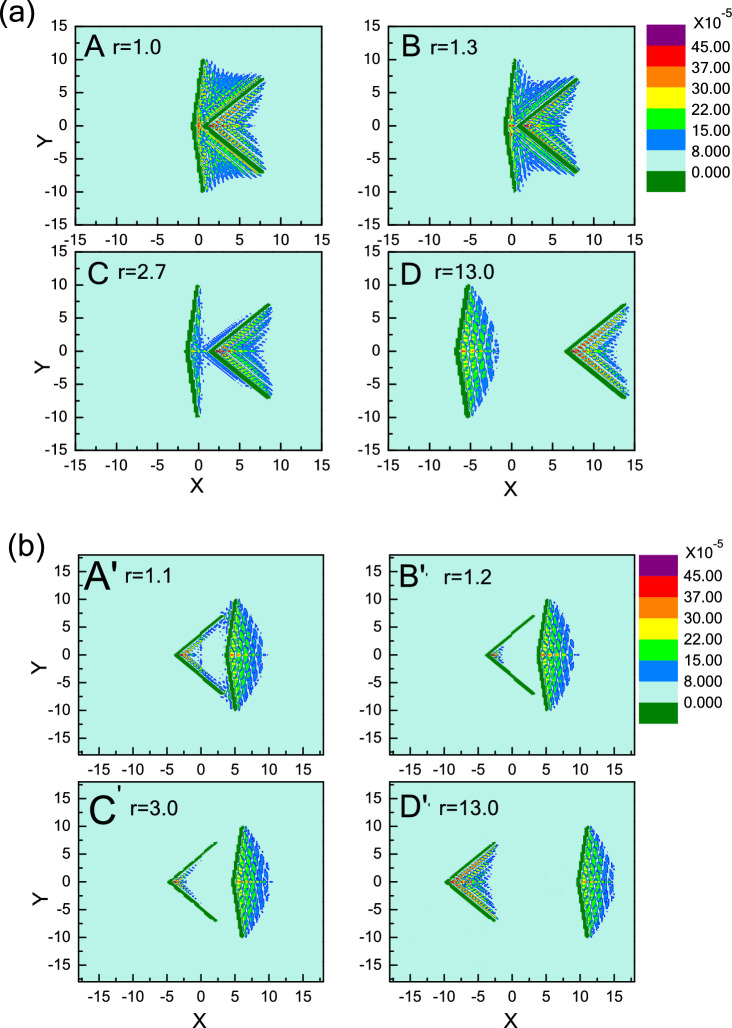
Density distributions of active particles for Model I- (**a**) and II-wedges (**b**) at different distances r.

Here, O_l(r) is denoted as the left side of the left (right) wedge and l(r)_O is defined as the right side of the left (right) wedge. From Fig. 2, it can be seen that there are two distinct transitions of effective force F(r) with increasing r, corresponding to repulsive-attractive-repulsive force (Model I in black), and only attractive force (Model II in red), respectively. To explore the physical mechanisms for the transition of effective force, we present the visual density distributions of active particles near the obstacles for different r, as shown in Fig. 3.

For Model I, Fig. 2 shows that the repulsive force (F(r) > 0) decreases with oscillation at first, and approaches to the minimum where attractive force (F(r) < 0) occurs between two asymmetric wedges, then arises gradually back to repulsive force (F(r) > 0) with the increase of distance r, and eventually remains constant. Figure 3a provides a strong support for explaining this non-monotonic repulsive-attractive-repulsive transition. As r = 1.0 corresponding to maximal repulsive force (point A in Fig. 2), the confinement region between two wedges is jammed with several dense layers of active particles forming a dynamic bridge structure due to the collective behaviour induced by the presence of a confined boundary^36^, as shown in Fig. 3a-A. Obviously, the repulsive force induced by dynamic particles bridge in confinement area is greater than the leftward force imposed on the right wedge, performing a net “repulsive force” between two asymmetric wedges. When the distance r is increased to r = 1.3 (point B in Fig. 2), the dynamic particles layer between two wedges becomes slightly thinner shown in Fig. 3a-B, which leads to a decrease in repulsive force between two wedges. Interestingly, for the case of 2.5 < r < 5.0, the effective force F(r) is reversed to be attractive. Take r = 2.7 for example shown in Fig. 3a-C, the dynamic bridge is broken, and the particle density in the inner contour of right wedge is greater than the confinement area, leading to an attraction between the two wedges. Finally, for r > 5.0, the interaction between two wedges rises gradually back to repulsive force (F(r) > 0), and eventually remains constant with the increase of distance r. For r = 13.0 shown in Fig. 3a-D, it can be seen that the distance is too far to have any overlap between the surrounding collective layers from two wedges, so the wedge is driven from their respective active particles gathered inside the wedge, where the particle number in the inside surface of left wedge is higher than that of right one. Moreover, the particle number difference would remain the same as further increasing r, so repulsive force would be constant.

Contrary to Model I, we exchange the position of these two wedges, named as Model II (θ_1_ = 90° and θ_2_ = 165°). The effective force F(r) between two wedges always is attractive, as shown in Fig. 2 (curve in red), which is completely different from Model I. For 0.8 < r < 3, the attractive force F(r) oscillates as r increases due to the oscillation of particle concentration between the inside and outside of the two wedges. These oscillation behaviours for effective forces are always observed in non-equilibrium system, especially for short-range distance^43,63^. For r = 1.1 shown in Fig. 3b-A′, a large cluster is formed near the entrance of the confinement area. As the repulsive part induced by internal particles is slightly less than the attractive part contributed by the particles in the inner contour of right wedge, a weak attraction occurs between two wedges. Compared with r = 1.1, as the distance r is increased to r = 1.2 shown in Fig. 3b-B′, the dynamic cluster disappears, and few particles can migrate into the cusp of the left wedge while most particles are captured by the right wedge. There is a maximal attractive force due to the great difference on the particle number captured by two wedges. For the case of r = 3.0, a few active particles enter the cusp of left wedge, reducing the particle concentration difference between two regions. That’s the reason why the attractive force F(r) for r = 3.0 is weaker than that for r = 1.2. Further, for r > 3.0, attractive force decreases firstly, and then keeps unchanged with increasing r. Obviously, as the distance is further enlarged, more particles are captured by the left wedge, which leads to the decrease of concentration difference between two regions. When concentration difference reaches the minimum, the attractive force arrives at the minimal value and then remains constant. For r = 13.0 as shown in Fig. 3b-D′, the wedge is driven from their respective active particles gathered inside the wedge. As the particle number in the inner part of right wedge is obviously higher than that of left wedge, the effective force is attractive. As further increasing r, the attractive force would be constant, which is contrary to the case shown in Fig. 3a-D.

**Figure 4:**
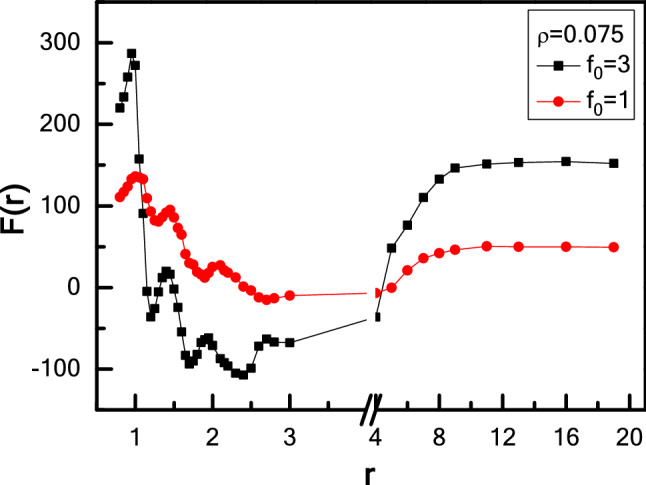
Effective force F(r) between two wedges as a function of r for Model I-wedges with various activities, *f*_*0*_.

In fact, the effective force between two asymmetric wedges is also influenced by the self-driving force $f_{0}$. As shown in Fig. 4, for a larger self-driving force $f_{0}$ = 3, the periodicity of oscillations is the same as in the case of $f_{0}$ = 1, while collisions of active particles with the wedges are more frequent, leading to a great oscillation amplitude of force F(r) compared to the case of $f_{0}$ = 1. Ni et al*.* found that both the strength and range of the effective forces between two parallel rods dramatically increase with the increase of self-driving force $f_{0}$. Here our results are also in good agreement with their results^63^. In addition, the effective force between two wedges is also related to the aspect ratio of active particle, p, and the detailed discussions are given in the Supplementary Information.

### Effects of particle density

**Figure 5:**
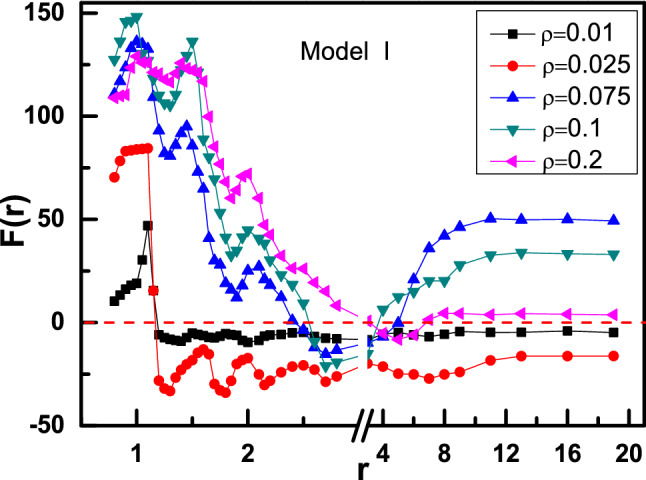
Effective force F(r) between two wedges as a function of r for Model I-wedges with different ρ.

**Figure 6:**
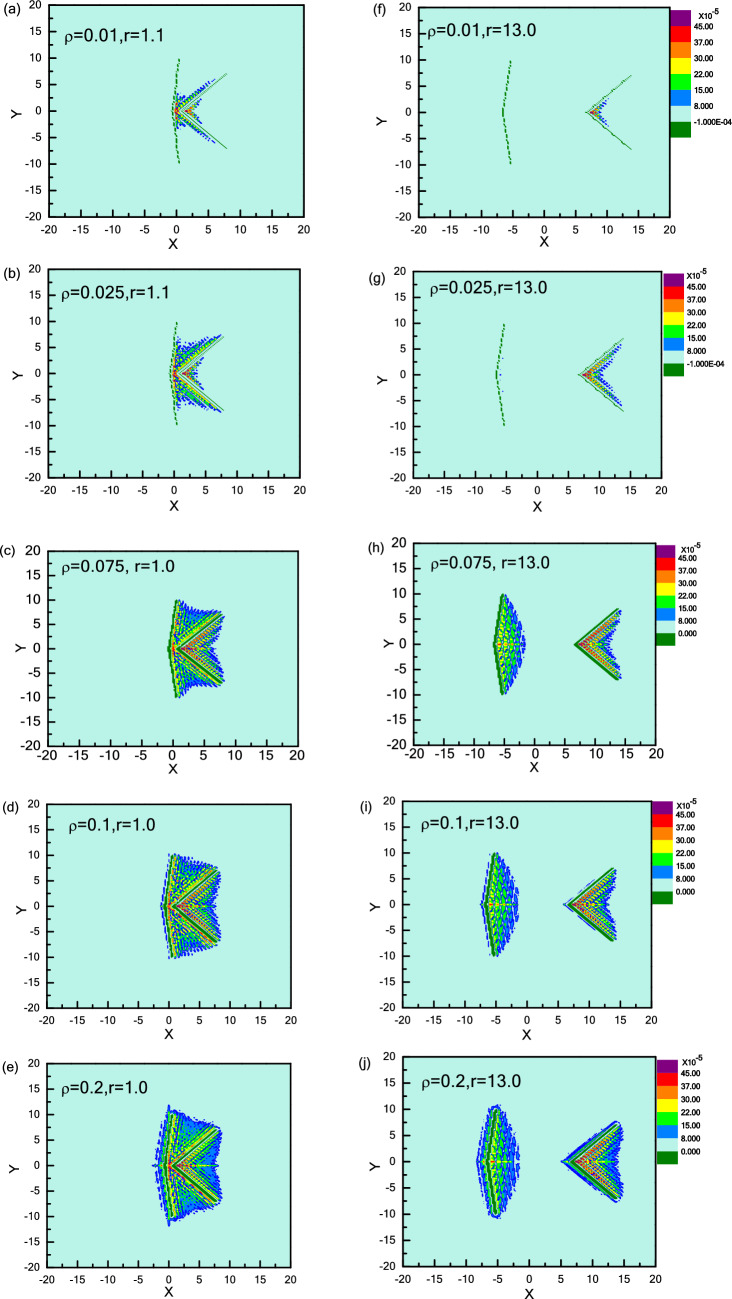
Density distribution of active particles for Model I-wedges at short-range distance (**a**) ~ (**e**) (r = 1.0) and long-range distance (**f**) ~ (**j**) (r = 13.0) with different ρ.

In this section, we will consider the effects of particle density on effective force between two asymmetric wedges. It can be found that the particle density ρ plays an important role in determining the direction and strength of the effective force. We first focus on Model I and show the interaction between two wedges versus r for different particle densities ρ, as shown in Fig. 5. In detail, for ρ = 0.075, 0.1, and 0.2, effective force F(r) experiences a repulsive-attractive-repulsive transition as r increases, while it undergoes the repulsive-attractive transition for low densities of ρ = 0.01 and 0.025. For all ρ, the effective forces F(r) oscillate firstly at short-range distance, and then achieve stably at long-range distance. For short-range distance, we pick the first peak of effective force F(*r*) curves for different ρ to display the density distribution of active particles in the left column of Fig. 6. For a low density of ρ = 0.01, as shown in Fig. 6a, a dynamic particle bridge forms between two wedges while only a few particles are captured by the inner contour of the right wedge, inducing the two wedges to repel with each other. With increasing particle density from ρ = 0.01 to 0.025, 0.075, and even 0.1, as shown in Fig. 6b–d, more particles swarm into the confinement area between two wedges for high density. The difference of particle concentration between inside and outside of two wedges increases, so the repulsive force between two wedges gets larger. Interesting, for a higher density of ρ = 0.2, the effective force F(*r*) between two wedges decreases. It indicates as the inner area of each wedge has reached saturation, further increasing ρ instead reduces the particle concentration difference between the inside and outside of one wedge and then weakens the effective repulsive force F(*r*), see Fig. 6e.

For long-range distance, we choose r = 13.0 to compare and the results are shown in the right column of Fig. 6. As ρ = 0.01, Fig. 6f shows that a few of particles are preferentially gathered in the cusp of the right wedge, while almost no particle is captured by the left wedge, therefore, interaction force between two wedges is weakly attractive. Increasing particle density from ρ = 0.01 to 0.025, as shown in Fig. 6g, more particles are found in the cusp of right wedge, and still only few particles are located in the inside surface of left wedge. The difference of particle concentration between two regions gets larger, so that the attractive force is strengthened. For ρ = 0.075, as shown in Fig. 6h, both two wedges can capture enough active particles and the collective behaviour reaches saturation. The number of particles captured by left wedge is more than that of right wedge, which induces a net repulsive force between two wedges. Further increasing ρ to 0.1 and 0.2 after saturation, more active particles can destroy the collective structure of the outer layer in the cusp of the left wedge, see Fig. 6i, j. Meantime, some active particles are also gathered in the left side of each wedge, which then weakens the effective repulsive force F(*r*).

**Figure 7:**
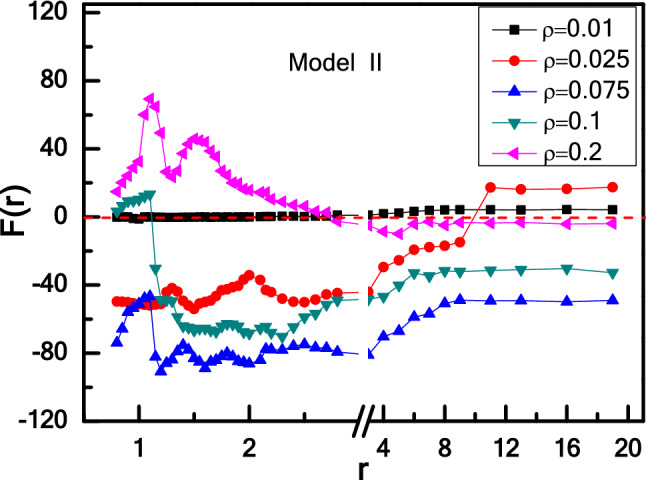
Effective force F(r) between two wedges as a function of r for Model II-wedges with different ρ.

For Model II, it is equivalent to exchange the position of the two wedges directly corresponding to Model I. As shown in Fig. 7, for ρ = 0.01, two wedges have no interaction for r < 3.0 as no particles are captured by wedge, and then repel with each other for r > 3.0 due to a few of particles are trapped preferentially by left wedge with θ_1_ = 90°. Increasing the particle density from ρ = 0.01 to 0.025, 0.075, 0.1 and even 0.2, the effective force oscillates and remains constant with r, which is similar to the case of Model I shown in Fig. 5. The oscillation of interaction at short-rang distance results from the oscillation of particle concentration difference between inside and outside of two wedges^43,62,63^. For ρ = 0.025, the attractive force F(r) increases compared with ρ = 0.01 at r < 3.0 because more particles are trapped by right wedge, and a transition from attraction to repulsion appears at r > 10.0. The transition from attraction to repulsion suggests that more particles are trapped by left wedge as increasing r. Further increasing ρ = 0.075 enhances the strength of the effective force, because the number of particles trapped by wedge increases with ρ increasing. For a higher particle density ρ = 0.1 and 0.2, the effective force decreases as ρ increasing. As the number of particles trapped by wedges has arrived at saturation, increasing ρ would reduce the difference of particle concentration between inside and outside of two wedges.

**Figure 8:**
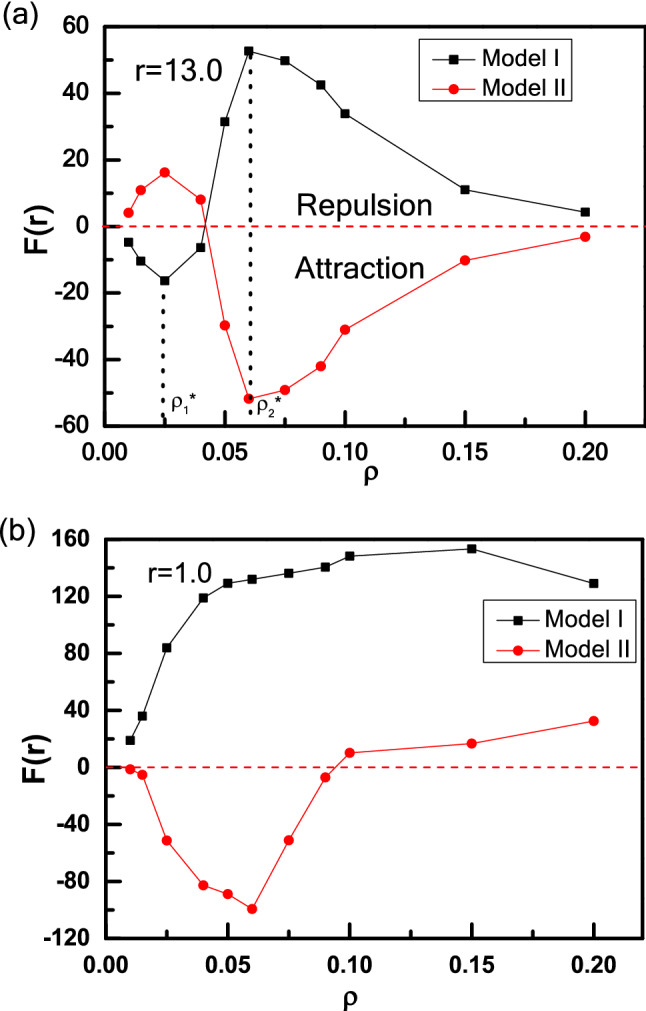
The repulsion-attraction transition for Model I- and II-wedges in an active bath induced by the density ρ at long-range distance (r = 13.0) (**a**) and short-range distance (r = 1.0) (**b**).

**Figure 9:**
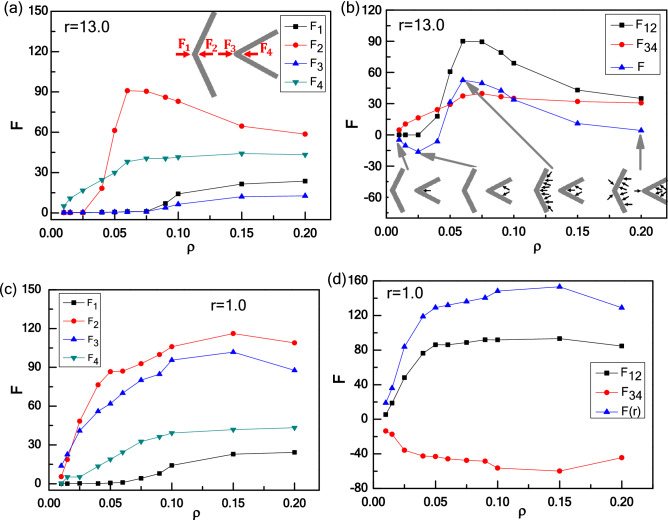
Independent force F acting on two wedges induced by active particles as a function of ρ for Model I-wedges: at long-range distance of r = 13.0 (**a**) and (**b**), and at short-range distance of r = 1.0 (**c**) and (**d**). Here, $F_{12} = F_{2} - F_{1} ,F_{34} = F_{4} - F_{3}$, and $F\left( r \right) = F_{12} - F_{34}$.

To explore the relationship between the effective force F(r) and the particle density in more detail, we calculate the effective force F(r) as a function of ρ for Model I- and II-wedges. We first discuss the case of long-range distance (r = 13.0) shown in Fig. 8a. For Model I, a transition from attraction to repulsion occurs with increasing ρ. There are two critical points (about at ρ_1_* ≈ 0.025 and ρ_2_* ≈ 0.06). The attractive force increases as the number of particles trapped by the right wedge with θ_2_ = 90° get larger with increasing ρ. Then the attractive force decreases to zero and reverse to repulsive force as more particles are captured by left wedge with θ_1_ = 165°, and the left wedge also gets saturation corresponding to the second critical point (ρ_2_* ≈ 0.06). Here two critical particle densities (ρ_1_* ≈ 0.025 and ρ_2_* ≈ 0.06) occur in the dilute regime, which are also consistent with the studies by Kaise et al.^36,66^. For a higher particle density (ρ = 0.1 and 0.2), more particles swim outside of two wedges, reduce the particle concentration difference between inside and outside of two wedges, and weaken the repulsive force between two wedges. Although the apex angle θ of the wedges plays a key role in determining the trapping efficiency of wedge, the total number of active particles trapped by the wedge also relies on the particle density ρ of active particles^36,66^. Symmetrically, for Model II, there is a transition from repulsion to attraction with increasing ρ. To clarify the underlying mechanism of this behaviour caused by particle density, we explore the independent force exerted by active particles of each side of two wedges, where the results are shown in Fig. 9. Figure 9a, b show that for low particle densities such as ρ < 0.025, there are more active particles gathered in the cusp of the right wedge than the left one, which leads to a fact that F_2_ is always close to 0, and F_4_ increases abruptly with particle density increasing. However, for ρ > 0.025, active particles are gathered massively in the cusp of the left wedge and F_2_ increases more greater than F_4_, which leads to the maximum of the difference between F_4_ and F_2_ at ρ = 0.060. As known, there are different collective abilities for the wedges with different angles of θ_1_ = 165° and θ_2_ = 90°. When ρ increases further, F_4_ increases slowly and the right wedge reaches saturation finally. However, F_2_ drops slowly instead because the overflowing particles would collide and even disturb the collective layer of the left wedge. Caprini and Marconi have investigated the effects of geometric confinement on the steady-state properties of a one-dimensional active suspension subject to thermal noise, and the random active force on the confining wall is studied both numerically, by integrating the Langevin governing equations, and analytically by solving the associated Fokker–Planck equation under suitable approximations^67^. They obtained a non-uniform density profiles because active particles accumulate near the wall, and the force exerted on the wall depends on the wall separation. Inspired by their work, we also calculate the independent force exerted on two sides of each wedge, explaining the attraction–repulsion transition of effective force between two asymmetric wedges. The force acting on the wedge originates from the average collision of active particles, and the underlying mechanism attributes the accumulation to the reduction of the active particles’ mobility in the presence of the wedges.

Then consider the case of short-range distance (r = 1.0) shown in Fig. 8b, differently from the long-range distance. For Model I, the repulsive force increases first and then decreases with increasing particle density ρ shown in Fig. 8b. Meantime, Fig. 9c, d show how the independent forces rely on the particle density ρ. With a low ρ, few particles are found in the confinement of two wedges. As increasing ρ, more particles flock into the confinement area, which is also reflected by the rapid growth of F_2_ and F_3_ shown in Fig. 9c, and leads to the increase of the repulsive force between two wedges. Once the number of particles trapped in the confinement achieves saturated, further increasing ρ would reduce the independent forces of F_2_ and F_3_ because more active particles can destroy the collective structures of active particles in the outer layer, which weakens the repulsive force a little. This result originates from the instability of trapping of active particle, which agrees well with the Kaiser’s results^36,66,68^. However, for Model II, two wedges attract first and then repel with each other with increasing ρ, different from the case of Model I. At a low particle density (ρ < 0.06), with increasing ρ, more and more particles are captured by right wedge, and the attraction gets stronger. As ρ > 0.06, more particles run into the confinement area, the attractive force is decreased to be zero and then reversed to repulsive force. From Fig. 8, we can conclude that by exchanging the position of the two asymmetric wedges, the transition of effective forces is symmetrical at long-range distance, while symmetry destruction occurs in short-range situations. Different behaviour for effective force between two asymmetric wedges at the long-range and short-range distances represents a typical ratchet phenomenon in active particle system in which it breaks the thermodynamical equilibrium, as well as it violates the left–right symmetry^38,51,52,54^.

### Effects of the apex angles of wedge

**Figure 10:**
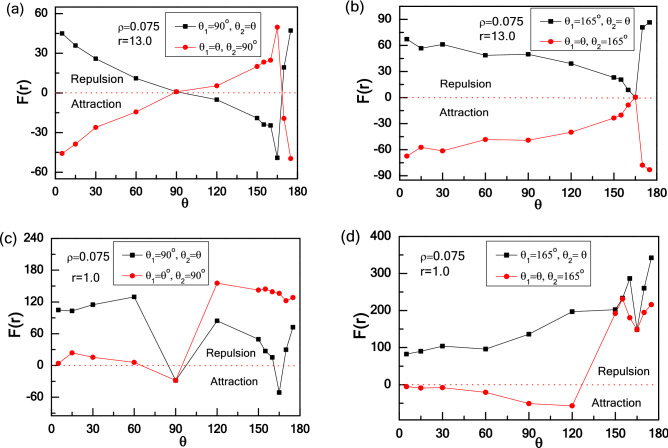
The repulsion and attraction force F(r) between two wedges in an active bath with different types of wedges: the apex angle of the one wedge is fixed: 90° (**a** and **c**) or 165° (**b** and **d**), and the other one is variable (0° ~ 180°).

**Figure 11:**
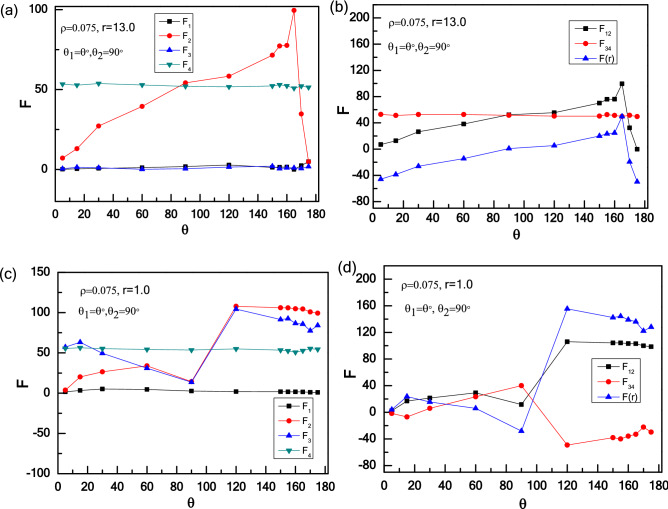
Independent force F acting on two wedges induced by active particles with the apex angle of the one wedge is fixed at 90°, and the other one is variable (0° ~ 180°): at long-range distance of r = 13 (**a**) and (**b**), and at short-range distance of r = 1.0 (**c**) and (**d**).

Finally, we investigate the effects of wedge shape on the interaction between two wedges. Here, we underline the asymmetric transition of the effective force at short-range distance and symmetric transition at long-range distance as a response to the interchange of two wedges positions, and the results are shown in Fig. 10. At long-range distance (r = 13.0) shown in Fig. 10a, the effective force displays symmetry for both cases: θ_1_ variable with θ_2_ = 90°, and θ_1_ = 90° with θ_2_ variable. For the former, an attraction–repulsion transition occurs as θ_1_ increases from θ_1_ = 0° to 180°. For a small angle (θ_1_ = 5°), most active particles are collected by right wedge, while hardly any particles are captured by left wedge, leading to an attraction between two wedges. Increasing θ_1_ from θ_1_ = 5° to 90°, the trap efficiency of left wedge improves, which would diminish the difference in the number of particles captured by two wedges and weaken the attraction. Similarly, increasing θ_1_ from 90° to 165°, more particles trapped by left wedge until the trap efficiency is maximized. Further increasing θ_1_, the trap efficiency of left wedge declines, which can lead to an attraction interaction between two wedges. As shown in Fig. 10b, one apex angles of two wedges is fixed at θ = 165°. For θ_1_ variable with θ_2_ = 165°, the interaction between two wedges is attractive overall, and the attraction increases and then decreases as θ_1_ increases. There is an optimal angle of θ_1_ = 165°, in which the trap efficiency reaches a maximum and two wedges have no interaction equivalently. In order to unveil the physical reason of the phenomenology observed, we monitor the independent force of each wedge attributed by the collisions between active particles and the wedge, and the results are given in Fig. 11. Figure 11a shows that F_1_, F_3_, and F_4_ keep unchanged, and only the force F_2_ acting on the inside surface of the left wedge varies as the apex angle increases, which leads to a non-monotonic behavior for the effective force F(r) between two wedges, as shown in Fig. 11b.

In addition, at short-range distance (r = 1.0) shown in Fig. 10c, the effective force shows two distinctive behaviours for both cases: θ_1_ = 90° with θ_2_ variable, and θ_1_ variable with θ_2_ = 90°. For θ_1_ = 90° with θ_2_ increasing from 5° to 60°, the repulsive force increases gradually. At θ_2_ = 5°, compared to this narrow angle, part of active particles is prioritized in the cusp of left wedge, which leads to a repulsive interaction between two wedges. With increasing θ_2_, particles bridges are formed gradually and a greater repulsion is produced. Especially for θ_2_ = 90°, few particles can swim into the confinement because the confinement is rather narrow, which results in an attraction. As 90° < θ_2_ < 120°, more particles can move into the confinement to enlarge the effective force. For 120° < θ_2_ < 165°, due to the trap efficiency of right wedge improving with the increase of the apex angle, the repulsive force decreases and even turns to be an attraction at θ_2_ = 165°. For θ_2_ > 165°, the trap efficiency of right wedge decreases, so that the repulsive force between two wedges gets larger. Conversely, for θ_1_ variable with θ_2_ = 90°, a weak repulsion increases first and then decreases at 5° < θ_1_ < 90°, and the effective force increases at 90° < θ_1_ < 120°, then slightly decreases as θ_1_ > 120°. With a very small angle of θ_1_ = 5°, particles cover the left side of the right wedge, so there is a weak repulsive force. Increasing θ_1_ from 15° to 90°, see Fig. 11c, d, F3 decreases as only few particles can stay the left side of the right wedge and F_2_ increases first, then decreases because the inside area of the left wedge gets larger, leading to a reduction of the effective force. For 90° < θ_1_ < 120°, the confinement can accommodate enough particles and strengthen the repulsive force. For θ_1_ > 120°, particles cannot stay in the confinement between two wedges stably for its large area, decreasing the repulsive force. Our result is consistent with the result of Kaiser et al.^36,37,66,68^, which is that the catching efficiency can be controlled by varying the apex angle of the wedge, and the particle density difference decides the direction and strength of the effective force between the two wedges.

For comparison, we also fix one apex angles of two wedges at θ = 165° and the other is variable, as shown in Fig. 10d. For θ_1_ = 165° with θ_2_ variable, the repulsive force increases at 5° < θ_2_ < 160°, then decreases at 160° < θ_2_ < 165°, and finally increases at θ_2_ > 165°. For 5° < θ_2_ < 160°, due to the confinement gets smaller, and particles bridges are formed leading to a greater repulsion. For 160° < θ_2_ < 165°, the confinement is too narrow to accommodate many active particles, producing a drop in repulsion. For θ_2_ > 165°, the trap efficiency of right wedge declines, so the repulsive force gets larger. For θ_1_ variable with θ_2_ = 165°, the attractive force increases during 5° < θ_1_ < 120°, due to the narrow confinement area and the high trap efficiency of right wedge. At 120° < θ_1_ < 155°, dynamic particle bridges are formed between two wedges, which enhances the repulsive force. At 155° < θ_1_ < 165°, the confinement is too narrow to accommodate many active particles, producing a drop in repulsion. At θ_1_ > 165°, particle bridges are formed again in the confinement between two wedges, enhancing the repulsion. Our results suggest the shape of wedge plays a crucial role in determining the trap efficiency, and there are three distinct states: no trapping at wide angles followed by a sharp transition towards complete trapping at medium angles and a crossover to partial trapping at small cusp angles^36^. We also explore the effective force of two parallel rods (i.e., θ_1_ = θ_2_ = 180°) in an active bath, and the results are shown in Supplementary Figure S2. Our result is consistent with the Kneževič’s work^62^, and some discussions are given in the Supplementary information. As a result, by exchanging the position of two asymmetric wedges, the transition of effective forces is symmetric for the long-range distance, while it is asymmetric for the short-range case. The dramatic collective response of active particles to the exchange of two wedges is noteworthy and never discovered in the passive system. We further emphasize the conversion of the interaction orientation is a collective, nonequilibrium behaviour.

## Conclusions

Using computer simulation, we studied the effective interaction between two asymmetric wedges exposed to a bath of active particles. The effective force between two wedges is strongly related to the relative position of two wedges, the wedge-to-wedge distance r, the particle density ρ, as well as the apex angle of wedge θ (θ_1_ or θ_2_). For a set of fixed apex angles with θ_1_ = 165° and θ_2_ = 90°, by only exchanging the position of the two asymmetric wedges, the left–right asymmetry due to this exchange and the self-driven behaviour of active particles together cause the two distinct transitions of effective force between two wedges. Further, with the exchange of the two wedge positions, whether it's changing particle density ρ or apex angle (θ_1_ or θ_2_), it is found that the effective force shows asymmetry for short-range distance between two asymmetric wedges, while it has symmetry for the long-range case. Our results further reveal that the effective force transition from attraction to repulsion is attributed to a collective, nonequilibrium effect and the self-driven behaviour of active particles. Our investigations may pave a novel way to applications for the design of tunable interactions by using active matter.

## Supplementary information

Supplementary information.
