# Supplementary material for: An attraction–repulsion transition of force on two asymmetric wedges induced by active particles
Source: Sci Rep. 2020 Jul 16;10:11702. doi: 10.1038/s41598-020-68677-w (PMC7367348; doi:10.1038/s41598-020-68677-w)
Supplement: Supplementary file 1 — Supplementary information. [file 41598_2020_68677_MOESM1_ESM.docx]

**Supplementary Information**

**An attraction-repulsion transition of force on two asymmetric wedges induced by active particles**

Ke Li, Fuchen Guo, Xiaolin Zhou, Xianghong Wang, Linli He, Linxi Zhang

**S1. Effects of the aspect ratio of active particles on effective force**

To explore the effects of the aspect ratio of active particles on effective force F(r), we have performed numerical simulations with different aspect ratios p in the case of Model I as shown in Figure S1. In Figure S1, we observe a similar damped oscillation at short-range distance and a repulsion interaction at long-range distance for different p. As r<3, active disks with p=1 have a larger oscillation amplitude of F(r) compared to the case of p=2, because more disks can be gathered near the wedges. Meanwhile, for r>8, more disks can be trapped by the left wedge, which leads to a larger repulsion interaction between two wedges at long-range distance. Therefore, the aspect ratio of active particles (p) does affect the effective force between two asymmetric wedges, which is consistent with previous result.^1^

**Figure S1**. Effective force F(r) between two wedges as a function of r for Model I-wedges with different aspect ratios of active particles, p. Here ρ=0.075.

**S2. Effective force between two rods immersed in an active particle bath**

Particularly, we also focus on the case of two parallel rods ( i. e., θ_1_=180°, θ_2_=180°). As shown in Figure S2, an oscillating attractive and repulsive force between two parallel rods is observed in the short-range distance, which is in agreement with previous results^2,3^. This behavior comes from the competition between the force exerted by active particles on the outside surface of two rods, and the formation of particle layer in the confinement of two rods. The oscillations of F(r) vanish until the separation r=3 and have a period of 0.5, which is roughly the same as the characteristic length of active particle. Obviously, for r>10, there is no interaction between two rods. In this case, partial active particles can form on the perimeter of two rods uniformly and swim around it, which balance the difference of the forces acting on two rods, resulting in F(r)=0 for r>10. This behavior agrees well with the works of Ran and Milos^2,3^.

Figure S2. Effective force F(r) between two parallel rods ( i.e., θ_1_=180°, θ_2_=180°) as a function of r with ρ=0.075.

References

1. Hua, Y. F., Li, K., Zhou, X. L., He, L. L. & Zhang, L. X. An attraction–repulsion transition of force on wedges induced by active particles. *Soft Matter* **14,** 5205-5212 (2018).
2. Ni, R., Cohen Stuart, M. A. & Bolhuis, P. G. Tunable Long Range Forces Mediated by Self-Propelled Colloidal Hard Spheres. *Phys. Rev. Lett.* **114,** 018302 (2015).
3. Kneževič, M. & Stark, H. Capillary condensation in an active bath, *arXiv*:1908.10483v1.
